# Moderate relationships between NAA and cognitive ability in healthy adults: implications for cognitive spectroscopy

**DOI:** 10.3389/fnhum.2014.00039

**Published:** 2014-02-14

**Authors:** Tulpesh Patel, Jacqueline C. Blyth, Gareth Griffiths, Deirdre Kelly, Joel B. Talcott

**Affiliations:** ^1^Aston Brain Centre, School of Life and Health Sciences, Aston UniversityBirmingham, UK; ^2^Birmingham Children's HospitalBirmingham, UK; ^3^European Bioenergy Research Institute, Aston UniversityBirmingham, UK

**Keywords:** proton magnetic resonance spectroscopy, N-acetyl aspartate, cognition, IQ, processing speed, cognitive spectroscopy

## Abstract

**Background:** Proton Magnetic Resonance Spectroscopy (^1^H-MRS) is a non-invasive imaging technique that enables quantification of neurochemistry *in vivo* and thereby facilitates investigation of the biochemical underpinnings of human cognitive variability. Studies in the field of cognitive spectroscopy have commonly focused on relationships between measures of N-acetyl aspartate (NAA), a surrogate marker of neuronal health and function, and broad measures of cognitive performance, such as IQ.

**Methodology/Principal Findings:** In this study, we used ^1^H-MRS to interrogate single-voxels in occipitoparietal and frontal cortex, in parallel with assessments of psychometric intelligence, in a sample of 40 healthy adult participants. We found correlations between NAA and IQ that were within the range reported in previous studies. However, the magnitude of these effects was significantly modulated by the stringency of data screening and the extent to which outlying values contributed to statistical analyses.

**Conclusions/Significance:** ^1^H-MRS offers a sensitive tool for assessing neurochemistry non-invasively, yet the relationships between brain metabolites and broad aspects of human behavior such as IQ are subtle. We highlight the need to develop an increasingly rigorous analytical and interpretive framework for collecting and reporting data obtained from cognitive spectroscopy studies of this kind.

## Introduction

Parallel refinements in neuropsychological assessment and neuroimaging techniques now enable the neurophysiological basis of individual variation in cognitive ability to be probed with increasing methodological precision (Jung and Haier, 2007; Haier, 2009). Proton magnetic resonance spectroscopy (^1^H-MRS) provides non-invasive quantification of neurochemicals and their metabolites in pre-defined regions of tissue, with a typical spatial resolution on the order of cubic centimeters. Voxel size is limited by a trade-off between the signal-to-noise ratio (SNR) and tissue specificity. With smaller voxel sizes, specificity of voxel localization, and therefore tissue type, is increased, but SNR is reduced (Freeman, 2003).

The use of ^1^H-MRS as a prognostic and diagnostic tool is well established (Danielsen and Ross, 1999; Burlina et al., 2000; Tran et al., 2009; Lee et al., 2012). Changes in the ^1^H-MRS spectrum reflect general pathology, such as demyelination or ischemia, and is associated with an array of neurological conditions, such as brain tumors (Hollingworth et al., 2006; Hou and Hu, 2009) multiple sclerosis (Gonzalez-Toledo et al., 2006; de Stefano and Filippi, 2007), traumatic brain injury (Marino et al., 2011), and neurodegenerative diseases such as Alzheimer's (Kantarci, 2007; Loos et al., 2010) and Parkinson's disease (Firbank et al., 2002; Rango et al., 2007). For some conditions, ^1^H-MRS has been used to identify a specific biomarker that aids diagnosis. For example, in Canavan's disease, a degenerative condition characterized by a progressive loss of myelin, there is a specific, robust increase in the N-acetyl aspartate (NAA) peak, reflective of the increased volume of this metabolite (Matalon et al., 1988; Namboodiri et al., 2006).

NAA is located primarily in pyramidal neurons, dendrites and axons, where it is involved in myelin lipid metabolism, neuronal osmoregulation and axon-glial signaling (Moffett et al., 2007). NAA is the most prominent and stable signal obtained with ^1^H-MRS in the human brain and provides a surrogate marker for the health and viability of neural tissue (Barker, 2001; Moffett et al., 2007), with concentrations in white matter reflecting the metabolic function of axons as well as the extent and efficiency of their myelination (Bjartmar et al., 2002). NAA may also enhance mitochondrial energy production from glutamate (Moffett et al., 2007) and function as a molecular water pump (Baslow, 2002; cf. Moffett et al., 2007), thereby increasing the speed and efficiency of neuronal signaling.

The putatively important role of NAA in neural tissue has been extended to investigations at more macroscopic levels of analysis, for example toward developing an understanding of the potential biochemical underpinnings of human cognitive ability. One general approach has been to measure NAA in cortical tissue in conjunction with psychometric measures of cognitive skills such as those provided by standardized IQ tests. In young, healthy adults, NAA in occipitoparietal white matter correlates to moderate effect with timed measures of neuropsychological performance, but less so with metrics of cognitive ability derived from non-timed tests (Jung et al., 1999a,b). NAA has also been demonstrated to be a predictor of moderate effect for Full-scale IQ (FSIQ), a construct derived from both timed and non-timed psychometric subscales (Jung et al., 2005).

Correlations between NAA and IQ variables vary not only with the IQ subscale assessed, however, but also with the cortical region and tissue type interrogated by the MRS voxel. For example, whilst small, positive correlations have been demonstrated between NAA concentration in anterior gray matter and verbal IQ, small, negative correlations have also been reported between NAA in posterior gray matter and measures of performance IQ (Jung et al., 2009). These patterns of result obtained with healthy adults suggest that NAA measured across different cortical regions co-varies with independent constructs of cognitive ability and with tissue type. This general pattern of effect has been observed in children (Yeo et al., 2000; Ozturk et al., 2009), adolescents (Gimenez et al., 2004; Aydin et al., 2012) and in older populations (Valenzuela et al., 2000; Ferguson et al., 2002; Ross et al., 2005; Charlton et al., 2007; Kochunov et al., 2010). In addition to these regionally specific effects, recent reports propose that whole brain NAA volumes may be positively correlated with both composite IQ measures and educational achievement in healthy adults (Glodzik et al., 2012), suggesting that both genetic and environmental variables mediate the relationship between NAA and cognitive variables.

Table 1 summarizes the results from published studies reporting the relationship between NAA and IQ variability. Of particular note is the substantial variability in the magnitude of the correlations between ^1^H-MRS-detectable NAA and targeted IQ constructs. These studies are also characterized by methodological differences in spectroscopic protocols, the neuroanatomical location of voxel placement, the choice of neuropsychological constructs tested, the population sampled and the data screening and statistical analysis strategies employed.

**Table 1:** **Summary of published investigations of the relationships between N-acetyl aspartate (NAA) and constructs of cognitive abilities in healthy samples**.

**Study**	***n***	**Age in years (*SD*)**	**Region(s)**	**Method**	**Key result**	**Correlation coefficient (*r*)**
Aydin et al., 2012	30	15.1 (0.75)	Genu, midbody and isthmus/splenium of corpus callosum	STEAM; Absolute concentrations	NAA in isthmus/splenium region positively correlated with PIQ^a^ and FSIQ^b^	0.57^a^ (*p* = 0.001), 0.55^b^ (*p* = 0.002)
Kochunov et al., 2010	38	67.9 (9.1)	Left/right FRO WM (forceps minor area)	PRESS; Absolute concentrations	NAA correlated with “psychomotor” cognitive processing speed	0.22 (*p* < 0.05)
Jung et al., 2009	63	23.7 (4.2)	Left/right, posterior/anterior G/WM	PRESS; Absolute concentrations	Lower right anterior GM NAA predicted VIQ^c^. Higher posterior GM NAA predicted PIQ^d^	0.01^c^ (*p* = 0.011), 0.01^d^ (*p* = 0.006)
Ozturk et al., 2009	51	12.3 (3.6)	Frontal WM, DLPFC, parietal WM, inferior parietal cortex, dorsal parietal cortex	Multisection spin-echo; Ratio to Cr	Left FRO WM NAA/Cr correlated with Purdue Pegboard right-hand raw scores^e^. Right FRO WM NAA/Cr correlated with SB-IV'Bead Memory' raw scores^f^	0.01^e^ (*p* = 0.047), 0.01^f^ (*p* = 0.032)
Charlton et al., 2007	78	58.2	Centrum semiovale WM	CSI; Absolute concentrations	NAA positively correlated with general composite measure of cognitive performance	0.33^¥^ (*p* < 0.01)
Jung et al., 2005	27	24.8 (5.9)	Left OP, left FRO and right FRO WM	PRESS; Absolute concentrations	Combination of higher left occipital WM and lower FRO WM NAA correlated with FSIQ in women	0.67 (*p* < 0.001)
Ross et al., 2005	59	70.8	Left frontal WM, midline OP GM	STEAM; Ratio to Cr and Ratio to water	Frontal WM NAA/H_2_O correlated with a composite measure representing speed of information processing, attentional function and visual memory	0.32 (*p* = 0.032)
Gimenez et al., 2004	21	14 (2.3)	Left medial temporal cortex	PRESS; Ratio to Cho	NAA/Cho related to free recall^g^ and recognition^h^ memory abilities	0.56^g^ (*p* = 0.008), 0.51^h^ (*p* = 0.018)
Pfleiderer et al., 2004	62	38.5 (15.4)	DLPFC, left ACC	STEAM; Absolute concentrations	NAA positively correlated with Verbal Intelligence in women in left DLPFC and left ACC	0.53 (*p* = 0.009)
Ferguson et al., 2002	88	65–70	Left parietal WM	PRESS; Ratios to Cr, Ratios to Cho, “adjusted metabolite values”	NAA/Cr correlated with Logical Memory, but effect due to Cr (Cr negatively correlated with Logical Memory)	0.24 (*p* < 0.05)
Valenzuela et al., 2000	20	72	Left OP and left FRO and WM	STEAM; Ratios to Cr	Frontal WM NAA/Cr correlated with executive-attentional cognitive ability	0.61 (*p* < 0.05)
Yeo et al., 2000	20	9.48 (1.67)	Right FRO WM	STEAM; Absolute concentrations	NAA positively correlated with Working Memory (Visual two-back test)	0.45 (*p* = 0.04)
Jung et al., 1999a	26	22 (4.6)	Left OP WM	STEAM; Absolute concentrations	NAA positively correlated with FSIQ^i^ (*p* < 0.001) and PIQ^j^	0.52^i^ (*p* < 0.001), 0.45^j^ (*p* < 0.001)
Jung et al., 1999b	45^†^	23 (4.9)	Left OP WM	STEAM; Absolute concentrations	NAA correlated with timed tasks^k^ to greater degree than general IQ^l^	0.66^k^ (*p* < 0.001), 0.59^l^ (*p* < 0.001)

*The principal findings from each study are summarized, along with key study characteristics and the associated correlation coefficients (r). The MRS acquisition method for each study is presented (STEAM, stimulated echo acquisition mode; PRESS, point-resolved spectroscopy) and whether absolute concentrations or relative (ratio) quantification was used. ACC, anterior cingulate cortex; DLPFC, dorsolateral prefrontal cortex; FRO, frontal; GM, gray matter; OP, occipitoparietal; WM, white matter*.

† *26 participants' data previously published in Jung et al. (1999a)*.

¥ *beta-weight*.

Coupled with the wide range of effect sizes reported for the sample of studies in Table 1, the substantial methodological variability between studies poses significant challenges for drawing strong inferences about the strength of the relationship between neurometabolites obtained with ^1^H-MRS and IQ variables. This difficulty is compounded by an apparent multiple comparisons problem that arises when studies have measured neurometabolites over multiple brain regions, and alongside a battery of intelligence measures, without detailing specific planned statistical comparisons *a priori*. Such studies may be particularly susceptible to inflated Type 1 error, resulting from the potentially large number of statistical tests generated from crossing multiple levels of different independent variables.

The aim of the current study was to assess the strength and significance of the relationship between ^1^H-MRS-detectable NAA volumes and cognitive ability, and specifically the different relationships proposed between timed and untimed IQ subscales (Jung et al., 1999a,b). The NAA resonance within white matter likely reflects both the metabolic function of the neuronal axons as well as the extent and efficiency of their myelination. Correspondingly, given NAA's putative regulatory role in neurophysiological processing speed—which may be a function of its important regulatory role within myelin lipid synthesis—we predicted strong associations between volumes of NAA in frontal (FRO) white matter and the Information Processing Speed (IPS) index from the Wechsler Adult Intelligence Scales (WAIS; Wechsler, 1997b). Furthermore, we predicted that the correlation between NAA and IPS would be stronger than that found for general cognitive ability [indexed by the WASI full-scale IQ (FSIQ)]. As a control condition, we measured NAA in voxels in occipitoparietal (OP) cortex, for which the same pattern of strong covariance with IQ measures was not predicted.

## Materials and methods

### Ethics statement

Written, informed consent was obtained from all participants under a protocol consistent with the tenets of the Declaration of Helsinki and with the approval of Aston University's Institutional Review Board and the Black Country Research Ethics Committee in the United Kingdom (08/H1202/38).

### Participants

We recruited 44 healthy volunteers (29 females) from the local population and from the university student body (mean age: 21.1, *SD* = 3.5). Participants were screened prior to testing to exclude the presence of probable neurological dysfunction, including previous serious brain injury, history of learning disability, neurological disease, psychiatric diagnosis or current use of psychoactive medication. The data from four participants were excluded at the outset due to poor-quality spectra resulting from susceptibility or motion artifact. Forty participants (27 females) were therefore included in the final analyses. The sample provides the study with statistical power in excess of 70% to detect moderate correlations of 0.4 and above, with statistical significance evaluated at an alpha level of 0.05 (Friedman, 1968).

### Neuropsychological assessment

The neuropsychological test battery comprised standardized indices of cognitive ability including IPS (Digit-Symbol Copy and Symbol Search from the WAIS) and FSIQ from the Wechsler Abbreviated Scales of Intelligence (WASI) (Wechsler, 1997a). All neuropsychological tests were completed in a single session and within 1 week of the neuroimaging protocol.

### Neuroimaging

**Figure 1:**
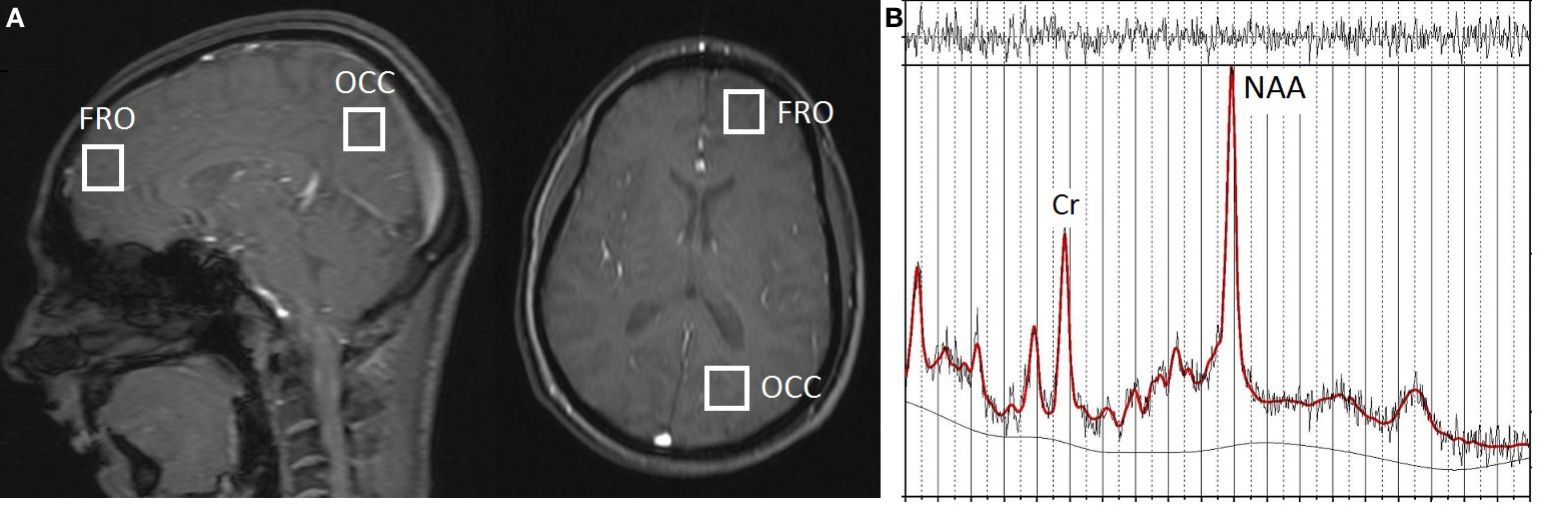
**(A)** Approximate localization of ^1^H-MRS voxels in frontal (FRO) and occipitoparietal (OP) cortex shown in the sagittal and axial planes with a 5-plane localizer (*TR* = 20 ms, *TE* = 5 ms, 10 slices at 5 mm thickness). **(B)** Representative ^1^H-MRS spectrum from a FRO voxel; Original data shown with the LModeled data overlaid (heavy line).

The ^1^H-MRS data were obtained with a Siemens 3T Trio scanner (Siemens Medical Solutions, Berkshire, UK) using standard acquisition software and a quadrature head coil. Single-voxel ^1^H-MRS was performed following localization of the volume (8 cm^3^) of interest using a 5-plane localizer (*TR* = 20 ms, *TE* = 5 ms, 10 slices at 5 mm thickness). For the left frontal (FRO) VOI, the voxel was positioned in the anterior cerebrum, centered superior to the lateral ventricles and avoiding overlap with non-neural sources and the corpus callosum. For the left occipitoparietal (OP) VOI, the voxel was placed in a region encompassing both occipital cortex and the inferior parietal lobule, with placement positioned to avoid overlap with the cerebellum (see Figure 1A). Manual voxel positioning maximized the contribution from white matter and minimized the contribution from gray matter. Automated shimming was followed by a STEAM pulse sequence (*TR* = 2000 ms, *TE* = 30 ms, 96 averages), including water suppression, for both frontal and occipitoparietal voxel acquisitions.

### ^1^H-MRS data screening

The data were screened in a two-stage process to ensure their reliability and validity prior to statistical analyses. In the first stage, spectra were visually inspected. High-quality spectra were identified by narrow, well-resolved peaks, a reasonably flat baseline and the presence of Hunter's Angle, a 45° angle connecting the major ^1^H-MRS peaks in normal tissue. Poor quality spectra were identified by distorted baselines and broadened spectral line widths (increased full-width, half maximum peak height). Online data screening allowed reacquisition of data if necessary, following adjustment of voxel positioning, and therefore maximized the probability of acquiring high-quality data from every participant.

To validate data quality, assess reliability, and quantify error variance introduced by scanner-related factors such as that caused by field inhomogenieties, data from two successive scans of the same voxel were obtained and averaged for each participant. Intraclass correlation coefficients across these two estimates were 0.947 and 0.910 for the FRO and OP voxels, respectively, demonstrating a high degree of test-retest reliability. The coefficient of variance within each voxel (9% in FRO and 8% in OP) was consistent with spectroscopic data published previously (Ross et al., 2005).

All data were analyzed with LCModel (Provencher, 2001), a software package that provides spectral quantification and metabolite detection optimized for short echo time spectroscopy. A representative ^1^H-MRS spectrum acquired from FRO voxels, showing original data with the modeled data (using LCModel) overlaid, is shown in Figure 1B.

**Figure 2:**
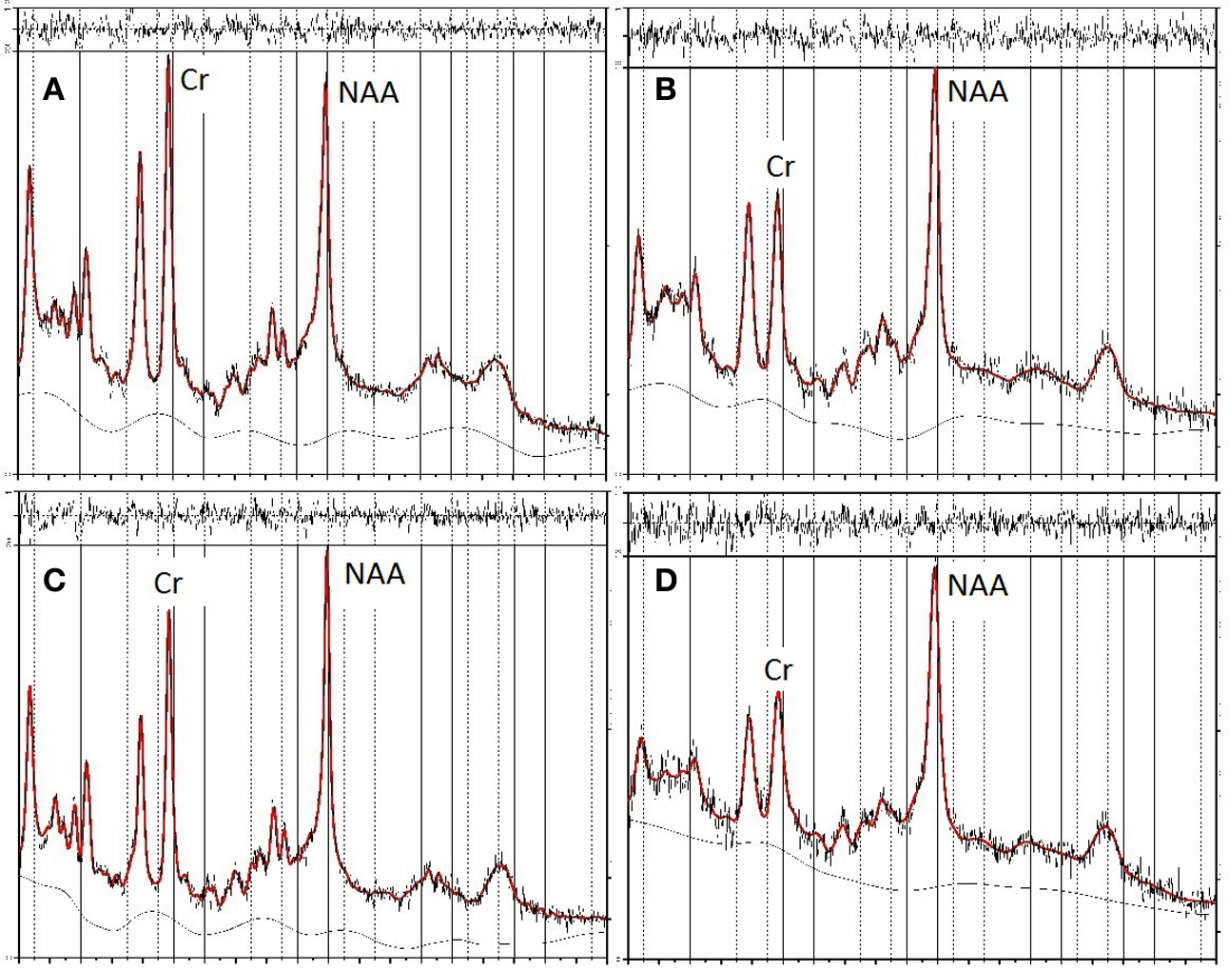
**LCModel spectra of unscreened data showing minimum and maximum values for NAA in frontal (FRO) and occipitoparietal (OP) voxels**. The top two panels show the spectra for minimum **(A)** and maximum **(B)** NAA/Cr concentrations in FRO voxels, the bottom two panels show the spectra for minimum **(C)** and maximum **(D)** NAA/Cr concentrations in OP voxels.

In the second stage of screening, data were excluded if metabolite values had a %SD (the estimated standard deviation, expressed as a percentage of the estimated concentration) exceeding 20%, the approximate criterion for acceptable reliability suggested by Provencher (2008). Three data sets were excluded by this criterion. Figure 2 shows the LCModel spectra for the maximum and minimum NAA/Cr values for FRO and OP voxels of the unscreened data. These data fulfilled the initial basic screening criteria for reliability and validity, as described above, but were subsequently excluded from data analyses as statistical outliers.

## Results

All analyses reported here adopt the standard convention of expressing NAA as a ratio to Cr (NAA/Cr). A summary of the descriptive data for the spectroscopic and psychometric measures is provided in Table 2. Mean metabolite ratios to Cr were consistent with those reported previously (Valenzuela et al., 2000; Ozturk et al., 2009). FSIQ scores were available for 31 participants and IPS scores for all 40 participants. MRS data for OP cortex was obtained for all 40 participants and for 36 participants for FRO volumes.

**Table 2:** **Descriptive statistics summarizing cognitive and ^1^H-MRS data for the sample**.

	**Mean (*SD*)**	**Min.–Max**.
Age (years)	21.2 (3.5)	18–34
FSIQ (SS)^*^	112.7 (12.3)	85–153
IPS (SS)	108.4 (13.0)	88–140
OP NAA/Cr	1.61 (0.21)	0.78–1.88
FRO NAA/Cr	1.56 (0.25)	0.42–1.95

SS, scaled score (mean = 100; SD = 15); FSIQ, Full-scale IQ, IPS, Information Processing Speed; OP, occipitoparietal; FRO, frontal;

* *n = 31 for this variable*.

No significant differences in NAA/Cr were observed between FRO and OP voxels overall [*t*_(35)_ = 1.86, *p* = 0.07]. Similarly, there were no main effects of sex on NAA/Cr in either region [OP: *t*_(36)_ = 2.78, *p* = 0.94; FRO: *t*_(34)_ = 0.02, *p* = 0.34].

Table 3 presents bivariate correlations between the spectroscopic and IQ variables. Here the data are presented in two complementary ways. First, the values obtained from all participants are included in the analyses. Figure 2 shows the LCModel spectra of the minimum and maximum NAA/Cr values in FRO and OP voxels for this unscreened data (see Table 2). Second, three univariate outliers, whose NAA/Cr value for either the FRO or OP voxel was 2SD or more away from the overall group mean, were removed from the analysis.

**Table 3:** **Correlations between NAA/Cr and psychometric measures of cognitive function in frontal (FRO) and occipitoparietal (OP) voxels**.

			**Parametric**		**Non-parametric**	
			**FSIQ**	**IPS**	**FSIQ**	**IPS**
Unscreened	FRO	*n*	31	40	31	40
		NAA/Cr	0.13	−0.19	−0.01	−0.25
	OP	*n*	31	40	31	40
		NAA/Cr	0.03	0.07	−0.06	−0.12
Screened	FRO	*n*	28	37	28	37
		NAA/Cr	−0.04	−0.29	−0.1	−0.26
	OP	*n*	28	37	28	37
		NAA/Cr	−0.12	−0.26	0.05	−0.25

*Abbreviations as for Table 2. Screened and unscreened data analyzed with non-parametric (Spearman's Rho) and parametric (Pearson Product Moment) correlations for comparison. 3 outlier values (> ± 2SD from cohort mean) excluded. Max r = −0.29 (p = 0.078) for Pearson correlation between FRO NAA and IPS*.

The unscreened data did not consistently satisfy distributional assumptions of normality (Shapiro–Wilk *W* >0.05) and were therefore analyzed using non-parametric tests of association. In contrast, the distribution of the screened data did not deviate significantly from normality and were analyzed using parametric correlations. The relationships between IPS or FSIQ constructs and NAA/Cr were neither strong, nor statistically significant, a pattern that was consistent across both OP and FRO voxels and both parametric and non-parametric analyses (see Table 3).

## Discussion

Changes in neurometabolite concentrations, particularly for NAA, have been linked to psychometric and performance variables across a range of disorders of cognitive function, including acute traumatic brain injury, and more chronic conditions such as schizophrenia and Alzheimer's disease (Danielsen and Ross, 1999; Burlina et al., 2000; Ross and Sachdev, 2004; Tran et al., 2009; Lee et al., 2012). This evidence has motivated investigations of normal populations to assess the ability of cognitive spectroscopy paradigms to identify potential biomarkers for variability in human intellectual function and achievement. Several studies have reported statistically significant correlations between NAA volumes and broad measures of cognitive skill, with small to moderate correlations typically found in samples of putatively normal populations (see Table 1).

In our study, we found that quantitative estimates of NAA in cortex did not correlate strongly or significantly with standardized measures of IQ constructs. The small correlations we obtained between NAA concentration and measures of cognitive function echo other findings that have reported weak associations between IQ and neurometabolite variables in healthy cohorts (Friedman et al., 1999; Shim et al., 2001; Filippi et al., 2002; Gimenez et al., 2004). However, they also contrast with other demonstrations of comparatively higher correlations between these constructs obtained with similar research designs (Jung et al., 1999a,b, 2005).

Based on the principal findings of the 14 studies included in Table 1, the average correlation between NAA and various measures of cognitive ability is 0.39 with a standard deviation of 0.23. The large standard deviation relative to the mean suggest the presence of inhomogeneous effects found across the population of published studies in this area. We suggest that this variability resulted at least in part from research design and methodological features that differed across studies. We therefore interpret the results of the present investigation within the context of these inter-study differences.

### Research design issues

#### Age

Previous studies employing cognitive spectroscopy of healthy populations have varied substantially in the age of the population sampled (see Table 1). Although we observed no significant correlations between NAA/Cr and age for either FRO or OP voxels in our study, the mean age and standard deviation of the participants (see Table 2) was similar to that obtained from other investigations of young, healthy adults, some of which have reported strong and significant IQ/neurometabolite correlations (Jung et al., 1999a,b, 2005). Whilst previous reports suggest the presence of age-related neurometabolic changes across the lifespan (Pouwels et al., 1999; Angelie et al., 2001), there appears to be no strong systematic relationship between the age of the study sampled and the strength of the IQ/neurometabolite correlations (*r* = 0.15, *p* = 0.55; see Table 1). Our interpretation of these data is that age of the cohort does not account for substantial variability in the strength of correlations between NAA and IQ in the cohort of previously published studies.

#### Sex

In our data set, we observed no main effect of sex on NAA/Cr values (echoing the findings of Charles et al., 1994; Pouwels and Frahm, 1998; Komoroski et al., 1999; Gimenez et al., 2004; Safriel et al., 2005), nor did we observe sex differences in the strength of correlations between NAA and cognitive measures in either FRO or OP regions. Few studies have reported sex differences in the relationship between NAA and cognitive ability. Two of the 14 studies in included in Table 1 (Ferguson et al., 2002; Aydin et al., 2012) studied only males. Pfleiderer et al. (2004) observed that NAA in the DLPFC (*n* = 52; 58% males) and in the left anterior cingulate cortex (*n* = 62; 65% males) was uniquely positively correlated with vocabulary scores (*r* = 0.53 and 0.56, respectively). Jung et al. (2005) observed that occipitoparietal NAA strongly correlated with FSIQ scores in women (adjusted *r*^2^ = 0.82), but not men (adjusted *r*^2^ = 0.35; *n* = 27; 63% male). When groups divided by sex were reasonably well-matched (*n* = 63; 54% male), Jung et al. (2009) found similar correlations for male and female participants (*r* = 0.34 and *r* = 0.24), specifically between right posterior gray matter NAA and Performance IQ. Jung et al. acknowledged that their study was underpowered for analysing potential sex differences in the context of other potentially significant factors such as the region and tissue type examined. The same limitation applies to our study, in which females were overrepresented.

#### Region

We observed no significant differences in NAA/Cr across cortical regions in our analyses, a result that is in accordance with some studies (for example, Ozturk et al., 2009), but which contrasts with others that have observed significant inter-regional differences using both single (Minati et al., 2010) and simultaneous acquisitions of multiple MRS voxels (Angelie et al., 2001; Maudsley et al., 2009). While the NAA/Cr ratios, both for our study and for those previously published (see Table 1) are consistent with normative data (Danielsen and Ross, 1999), it would not be surprising if the correlations between neurometabolites and cognitive function varied according to the cortical region investigated, based on knowledge of the functional specialization of the cerebral cortex. It was for this reason that we obtained measures from FRO voxels, given the importance of this region for the cognitive functions assessed with the WAIS measures (Baddeley, 1996; Duncan et al., 2000), and a control volume in OP cortex, for which we would not predict the same magnitude of relationship.

#### Tissue type

The typically large spatial extent of the voxel from which data is acquired in MRS studies makes it difficult to obtain measures from homogeneous tissue. However, decreasing the spatial extent of the size of the voxel leads to a corresponding decrease in the signal to noise ratio (Freeman, 2003). The extent to which MRS samples gray or white matter can modulate both the metabolite values obtained (Wang and Li, 1998; McLean et al., 2000; Wiedermann et al., 2001) and the pattern of NAA/IQ relationships (Jung et al., 2009). The precision gained from obtaining measures from homogeneous tissue, or after correcting for tissue inhomogeneity, is a reasonable aspiration for future studies. Of the studies included in Table 1, Charlton et al. (2007) is the exception in that they excluded voxels containing less than 75% white matter. They acknowledged, however, the inevitable reduction in sample size (and correspondingly in nominal statistical power) that potentially results from applying such *post-hoc* screening criteria in research studies.

### Methodological issues

#### Assessments of cognitive ability

Previous studies have typically adopted one of three general strategies to assess cognitive ability psychometrically: (1) broad, non-specific measures of cognitive ability, such as those obtained by standardized assessments of general intelligence [e.g., FSIQ, Verbal IQ and Performance IQ from standardized test batteries (Wechsler, 1997a,b)]; (2) data reduction techniques to derive composite IQ scores for use as dependent variables (Valenzuela et al., 2000; Ross et al., 2005; Kochunov et al., 2010; Glodzik et al., 2012); or (3) measuring more specific cognitive domains such as working memory (Yeo et al., 2000) or IPS (Jung et al., 1999b). For correlations with NAA, studies (*n* = 9) that used broad-based measures such as FSIQ or a composite score derived by factor analysis have an average correlation coefficient (Spearman's Rho) of 0.44 (*SD* = 0.23). In contrast, those studies (*n* = 4) reporting findings for specific cognitive measures yield an average correlation of 0.30 (*SD* = 0.25) (see Table 1). A minority of studies have framed more specific hypotheses, for example, proposing sex differences in NAA and verbal processing ability (Pfleiderer et al., 2004) or hemispheric specificity with respect to verbal working memory and motor speed (Ozturk et al., 2009). The associated correlation coefficients for these relationships is characteristically variable (*r* = 0.67 and 0.01, respectively).

In our study, we opted for a hybrid approach, assessing both a broad measure of IQ and the IPS index, which taps a smaller set of abilities in mental and motor speed (Groth-Marnat et al., 2000). Given NAA's putative regulatory role in neurophysiological processing speed (Jung et al., 1999a,b), we predicted, although did not find, strong positive associations between levels of NAA in frontal (FRO) cortex and WAIS IPS scores (Wechsler, 1997b) (see Table 3).

The methodological approach to data analytic strategy should be directly informed by the hypotheses adopted *a priori* (or lack thereof), which then governs and constrains the choice and range of psychometric assessments applied and how they are treated in statistical analyses. Given the potentially diffuse role of NAA in brain function, broader-based measures of cognitive function may provide the most robust correlations because both MRS-detectable NAA and psychometric scores provide by proxy measurement of underlying and potentially correlated constructs.

#### Metabolite quantification

Published studies vary according to whether absolute concentrations of NAA are used or whether NAA values are expressed as a ratio to another neurometabolite (typically Cr or Cho). Expressing NAA as a ratio to another metabolite confers the advantage of correcting for potentially important unknown or uncontrollable, yet correlated, experimental factors, such as static (B0) and radio frequency (RF, B1) field inhomogeneity. Ratio values continue to be used, for example in studies of mild cognitive impairment (Nie et al., 2013; Targosz-Gajniak et al., 2013), where cognitive spectroscopy research is particularly productive. However, the validity of using such ratios may be compromised by the potentially under-conservative assumption of the stability of the reference metabolite (Li et al., 2003). For example, Cr values may change not only in disease states (Maniega et al., 2008) but also during the course of normal aging (Haga et al., 2009). An alternative strategy is to use one of a number of absolute quantification methods (Jansen et al., 2006), and in particular using the water signal as reference (Keevil et al., 1998). However, this method is also subject to limitations. The water fractions within gray and white matter differ, which necessitates precise tissue partitioning, a requirement most reliably obtained with voxels of small spatial extent and with post-acquisition verification of voxel positioning.

Despite these potential issues, our analysis of the studies in Table 1 did not identify any systematic relationship between the method of metabolite quantification adopted and the size and strength of the reported findings between IQ and NAA [*t*_(18)_ = 0.968, *p* = 0.35 for between group effects using absolute or relative quantification methods]. Whilst the quantification method does not seem to be a significant factor in this specific context, until a standard referencing procedure is widely adopted, comparing data across studies in different domains that differ in this variable will remain difficult (de Beer et al., 1995; Knight-Scott et al., 2003). Furthermore, investigating multivariate relationships using multiple measures of metabolite expressions, such as with both absolute values and ratios, may only compound difficulties in interpretation because this procedure increases greatly the number of potential statistical comparisons within a given study.

#### Data screening and analysis

The procedures used for data screening, coupled with the suitability of the statistical analysis strategy adopted, may critically underpin the validity of the findings gleaned from a given study. Differences between studies in Table 1 were also apparent in their analytical strategies, including procedures for data screening to ensure robust correlational data (Tabachnik and Fidell, 2001). Data validity depends on several factors, including the assumption of univariate and multivariate normality and how outlying values are handled in statistical analyses. To date, published investigations have employed highly variable procedures for data screening, with little homogeneity of practice in the methods of analysis applied, or in the presentation of data. Levels of screening range from general removal of poor quality spectra (Jung et al., 1999b; Ross et al., 2005), to exclusion of metabolite values exceeding a critical statistical cut-off, either following *post-hoc* quantification (Ferguson et al., 2002; Pfleiderer et al., 2004; Jung et al., 2005) or screening for voxel tissue composition, but not the metabolite values obtained (Charlton et al., 2007). Other studies (Jung et al., 1999a; Valenzuela et al., 2000; Yeo et al., 2000; Gimenez et al., 2004; Kochunov et al., 2010) have not reported details of the data screening procedures employed.

All of the studies in Table 1 employed parametric data analyses. As demonstrated in the present study, distributional normality of spectroscopy data cannot be assumed, yet previously published studies have not included information on the normality or distributional assumptions of their data. Analysis of our unscreened data with parametric measures (which were not justified in this case), results in significant correlations between NAA and IQ variables on the order of *r* = 0.40, a finding similar to those previously reported (i.e., the average correlation of the studies in Table 1 is 0.39). Given that the potential impact of outliers and non-Gaussian distributions of data appear to modulate the pattern of results, we suggest that the extent and nature of data screening employed should be routinely reported in cognitive spectroscopy studies.

Lack of robust data screening of relatively small samples, combined with the large potential number of statistical comparisons, and a lack of specific comparisons identified *a priori*, can result in the inflated probability of Type 1 error. Correspondingly, analyses of the studies published to date (Table 1) suggest that as sample size increases, the strength of the correlation between NAA and IQ constructs tends to decrease (Spearman's Rho = –0.51; *p* = 0.025).

A key future consideration for studies in cognitive spectroscopy is to distinguish between statistically significant effects and ones that are statistically relevant, particularly for cases where effect sizes may not be large enough to survive statistical adjustments for multiple comparisons or were not identified as planned comparisons *a priori*. Whilst our initial sample size of 44 is relatively modest, it falls well within the range of published studies and is sufficiently large enough to detect moderate correlations between NAA and cognitive ability with adequate statistical power. The results of this quantitative review suggest that many studies in this area have been underpowered statistically to reliably detect subtle relationships between neurometabolite volumes and constructs of human cognitive ability. The subtlety of the relationships motivates the collection of larger samples of participants than have been typically obtained to achieve the statistical power necessary to reliably detect multiple significant effects and their interactions (Jung et al., 2009; Ozturk et al., 2009).

## Conclusions

^1^H-MRS provides a valuable and sensitive tool for quantifying neurochemistry *in vivo*. In this study of healthy adults, we adopted strict screening criteria to ensure robust, reliable, single-voxel metabolite data and observed statistically weak relationships between frontal cortex NAA and FSIQ and IPS. Although not significant statistically, the effect sizes obtained fell in the range of those reported in previously published studies. Whilst our null findings may be the result of several factors related to methodology and data screening procedures, the lack of a standard practice the reporting of data in the literature suggests that conclusions drawn from these types of studies still need to be tempered by consideration of the methodological framework employed. Despite the limitations, there is the potential for ^1^H-MRS to make a significant contribution toward bridging the gap from micro- to macroscopic levels of analysis that underpin brain-behavior relationships.

### Conflict of interest statement

This work was supported the Engineering and Physical Sciences Research Council and by a neuroimaging infrastructure grant from the Lord Dowding Fund. The funders had no role in study design, data collection and analysis, decision to publish, or preparation of the manuscript.

## References

[B1] Angelie E. Bonmartin A. Boudraa A. Gonnaud P. M. Mallet J. J. Sappey-Marinier D. (2001). Regional differences and metabolic changes in normal aging of the human brain: proton MR spectroscopic imaging study. AJNR Am. J. Neuroradiol. 22, 119–127 Available online at: http://www.ajnr.org/content/22/1/119.long 11158897 PMC7975557

[B2] Aydin K. Uysal S. Yakut A. Emiroglu B. Yilmaz F. (2012). N-acetylaspartate concentration in corpus callosum is positively correlated with intelligence in adolescents. Neuroimage 59, 1058–1064 10.1016/j.neuroimage.2011.08.114 21983183

[B3] Baddeley A. (1996). Exploring the central executive. Q. J. Exp. Psychol. 49A, 5–28 10.1080/027249896392784

[B4] Barker P. B. (2001). N-acetyl aspartate–a neuronal marker? Ann. Neurol. 49, 423–424 10.1002/ana.90 11310618

[B5] Baslow M. H. (2002). Evidence supporting a role for N-acetyl-L-aspartate as a molecular water pump in myelinated neurons in the central nervous system. An analytical review. Neurochem. Int. 40, 295–300 10.1016/S0197-0186(01)00095-X 11792458

[B6] Bjartmar C. Battistuta J. Terada N. Dupree E. Trapp B. D. (2002). N-acetylaspartate is an axon-specific marker of mature white matter *in vivo*: a biochemical and immunohistochemical study on the rat optic nerve. Ann. Neurol. 51, 51–58 10.1002/ana.10052 11782984

[B7] Burlina A. P. Aureli T. Bracco F. Conti F. Battistin L. (2000). MR spectroscopy: a powerful tool for investigating brain function and neurological diseases. Neurochem. Res. 25, 1365–1372 10.1023/A:1007660632520 11059807

[B8] Charles H. C. Lazeyras F. Krishnan K. R. R. Boyko O. B. Patterson L. J. Doraiswamy P. M. (1994). Proton spectroscopy of human brain: effects of age and sex. Prog. Neuropsychopharmacol. Biol. Psychiatry 18, 995–1004 10.1016/0278-5846(94)90125-2 7824764

[B9] Charlton R. A. McIntyre D. J. Howe F. A. Morris R. G. Markus H. S. (2007). The relationship between white matter brain metabolites and cognition in normal aging: the GENIE study. Brain Res. 1164, 108–116 10.1016/j.brainres.2007.06.027 17632090

[B10] Danielsen E. R. Ross B. (1999). Magnetic Resonance Spectroscopy Diagnosis of Neurological Diseases. New York, NY: Marcel Dekker

[B11] de Beer R. Bachert-Baumann P. Bovee W. M. Cady E. Chambron J. Dommisse R. (1995). Quality assessment in *in vivo* NMR spectroscopy: VI. Multicentre quantification of MRS test signals. Magn. Reson. Imag. 13, 169–176 10.1016/0730-725X(94)00092-H 7898276

[B12] de Stefano N. Filippi M. (2007). MR spectroscopy in multiple sclerosis. J. Neuroimag. 17(Suppl. 1), 31S–35S 10.1111/j.1552-6569.2007.00134.x 17425732

[B13] Duncan J. Seitz R. J. Kolodny J. Bor D. Herzog H. Ahmed A. (2000). A neural basis for general intelligence. Science 289, 457–460 10.1126/science.289.5478.457 10903207

[B14] Ferguson K. J. Maclullich A. M. Marshall I. Deary I. J. Starr J. M. Seckl J. R. (2002). Magnetic resonance spectroscopy and cognitive function in healthy elderly men. Brain 125, 2743–2749 10.1093/brain/awf278 12429601

[B15] Filippi C. G. Ulug A. M. Deck M. D. Zimmerman R. D. Heier L. A. (2002). Developmental delay in children: assessment with proton MR spectroscopy. AJNR Am. J. Neuroradiol. 23, 882–888 Available online at: http://www.ajnr.org/content/23/5/882.long 12006297 PMC7974746

[B16] Firbank M. J. Harrison R. M. O'Brien J. T. (2002). A comprehensive review of proton magnetic resonance spectroscopy studies in dementia and Parkinson's disease. Dement. Geriatr. Cogn. Disord. 14, 64–76 10.1159/000064927 12145453

[B17] Freeman R. (2003). Magnetic Resonance in Chemistry and Medicine. Oxford: Oxford University Press

[B18] Friedman H. (1968). Magnitude of experimental effect and a table for its rapid estimation. Psychol. Bull. 70, 245–253 10.1037/h0026258

[B19] Friedman S. D. Brooks W. M. Jung R. E. Chiulli S. J. Sloan J. H. Montoya B. T. (1999). Quantitative proton MRS predicts outcome after traumatic brain injury. Neurology 52, 1384–1391 10.1212/WNL.52.7.1384 10227622

[B20] Gimenez M. Junque C. Narberhaus A. Caldu X. Segarra D. Vendrell P. (2004). Medial temporal MR spectroscopy is related to memory performance in normal adolescent subjects. Neuroreport 15, 703–707 10.1097/00001756-200403220-00026 15094480

[B21] Glodzik L. Wu W. E. Babb J. S. Achtnichts L. Amann M. Sollberger M. Monsch A. U. (2012). The whole-brain N-acetylaspartate correlates with education in normal adults. Psychiatry Res. 204, 49–54 10.1016/j.pscychresns.2012.04.013 23177924 PMC3508436

[B22] Gonzalez-Toledo E. Kelley R. E. Minagar A. (2006). Role of magnetic resonance spectroscopy in diagnosis and management of multiple sclerosis. Neurol. Res. 28, 280–283 10.1179/016164106X98161 16687054

[B23] Groth-Marnat G. Gallagher R. E. Hale J. B. Kaplan E. (2000). The wechsler intelligence scales, in Neuropsychological Assessment in Clinical Practice. A Guide to Test Interpretation and Integration, ed Groth-Marnat G. (New York, NY: John Wiley & Sons, Inc.), 129–194

[B24] Haga K. K. Khor Y. P. Farrall A. Wardlaw J. M. (2009). A systematic review of brain metabolite changes, measured with 1H. Neurobiol. Aging 30, 353–363 10.1016/j.neurobiolaging.2007.07.005 17719145

[B25] Haier R. J. (2009). Neuro-intelligence, neuro-metrics and the next phase of brain imaging studies. Intelligence 37, 121–123 10.1016/j.intell.2008.12.006

[B26] Hollingworth W. Medina L. S. Lenkinski R. E. Shibata D. K. Bernal B. Zurakowski D. (2006). A systematic literature review of magnetic resonance spectroscopy for the characterization of brain tumors. AJNR Am. J. Neuroradiol. 27, 1404–1411 Available online at: http://www.ajnr.org/content/27/7/1404.long 16908548 PMC7977543

[B27] Hou B. L. Hu J. (2009). MRI and MRS of human brain tumors. Methods Mol. Biol. 520, 297–314 10.1007/978-1-60327-811-9_21 19381963

[B27a] Jansen J. F. Backes W. H. Nicolay K. Kooi M. E. (2006). 1H MR spectroscopy of the brain: absolute quantification of metabolites. Radiol. 240, 318–332 16864664 10.1148/radiol.2402050314

[B28] Jung R. E. Brooks W. M. Yeo R. A. Chiulli S. J. Weers D. C. Sibbitt W. L. Jr. (1999a). Biochemical markers of intelligence: a proton MR spectroscopy study of normal human brain. Proc. Biol. Sci. U.S.A. 266, 1375–1379 10.1098/rspb.1999.0790 10445292 PMC1690078

[B30] Jung R. E. Gasparovic C. Chavez R. S. Caprihan A. Barrow R. Yeo R. A. (2009). Imaging intelligence with proton magnetic resonance spectroscopy. Intelligence 37, 192–198 10.1016/j.intell.2008.10.009 19936275 PMC2780337

[B31] Jung R. E. Haier R. J. (2007). The Parieto-Frontal Integration Theory (P-FIT) of intelligence: converging neuroimaging evidence. Behav. Brain Sci. 30, 135–154 discussion: 154–187. 10.1017/S0140525X07001185 17655784

[B32] Jung R. E. Haier R. J. Yeo R. A. Rowland L. M. Petropoulos H. Levine A. S. (2005). Sex differences in N-acetylaspartate correlates of general intelligence: an 1H-MRS study of normal human brain. Neuroimage 26, 965–972 10.1016/j.neuroimage.2005.02.039 15955507

[B29] Jung R. E. Yeo R. A. Chiulli S. J. Sibbitt W. L. Jr. Weers D. C. Hart B. L. (1999b). Biochemical markers of cognition: a proton MR spectroscopy study of normal human brain. Neuroreport. 10, 3327–3331 10.1097/00001756-199911080-00014 10599840

[B33] Kantarci K. (2007). 1H magnetic resonance spectroscopy in dementia. Br. J. Radiol. 80, S146–S152 10.1259/bjr/60346217 18445744

[B34] Keevil S. F. Barbiroll B. Brooks J. C. W. Cady E. B. Canese R. Carlier D. J. (1998). Absolute metabolite quantification by *in vivo* NMR spectroscopy: II. A multicentre trial of protocols for *in vivo* localised proton studies of human brain. Magn. Reson. Imaging. 16, 1093–1106 9839993 10.1016/s0730-725x(98)00118-0

[B35] Knight-Scott J. Haley A. P. Rossmiller S. R. Farace E. Mai V. M. Christopher J. M. (2003). Molality as a unit of measure for expressing 1H MRS brain metabolite concentrations *in vivo*. Magn. Reson. Imag. 21, 787–797 10.1016/S0730-725X(03)00179-6 14559344

[B36] Kochunov P. Coyle T. Lancaster J. Robin D. A. Hardies J. Kochunov V. (2010). Processing speed is correlated with cerebral health markers in the frontal lobes as quantified by neuroimaging. Neuroimage 49, 1190–1199 10.1016/j.neuroimage.2009.09.052 19796691 PMC2789896

[B37] Komoroski R. A. Heimberg C. Cardwell D. Karson C. N. (1999). Effects of gender and region on proton MRS of normal human brain. Magn. Reson. Imag. 17, 427–433 10.1016/S0730-725X(98)00186-6 10195586

[B38] Lee M. R. Denic A. Hinton D. J. Mishra P. K. Choi D. S. Pirko I. (2012). Preclinical (1)H-MRS neurochemical profiling in neurological and psychiatric disorders. Bioanalysis 4, 1787–1804 10.4155/bio.12.129 22877223 PMC3922617

[B39] Li B. S. Y. Wang H. Gonen O. (2003). Metabolite ratios to assumed stable creatine level may confound the quantification of proton brain MR spectroscopy. Magn. Res. Imag. 21, 923–928 10.1016/S0730-725X(03)00181-4 14599543

[B40] Loos C. Achten E. Santens P. (2010). Proton magnetic resonance spectroscopy in Alzheimer's disease, a review. Acta Neurol. Belg. 110, 291–298 21305856

[B41] Maniega S. M. Cvoro V. Armitage P. A. Marshall I. Bastin M. E. Wardlaw J. M. (2008). Choline and creatine are not reliable denominators for calculating metabolite ratios in acute ischemic stroke. Stroke 39, 2467–2469 10.1161/STROKEAHA.107.507020 18617668

[B42] Marino S. Ciurleo R. Bramanti P. Federico A. De Stefano N. (2011). 1H-MR spectroscopy in traumatic brain injury. Neurocrit. Care 14, 127–133 10.1007/s12028-010-9406-6 20737247

[B43] Matalon R. Michals K. Sebesta D. Deanching M. Gashkoff P. Casanova J. (1988). Aspartoacylase deficiency and N-acetylaspartic aciduria in patients with Canavan disease. Am. J. Med. Genet. 29, 463–471 10.1002/ajmg.1320290234 3354621

[B44] Maudsley A. A. Domenig C. Govind V. Darkazanli A. Studholme C. Arheart K. (2009). Mapping of brain metabolite distributions by volumetric proton MR spectroscopic imaging (MRSI). Magn. Reson. Med. 61, 548–559 10.1002/mrm.21875 19111009 PMC2724718

[B45a] McLean M. A. Woermann F. G. Barker G. J. Duncan J. S. (2000). Quantitative analysis of short echo time (1)H-MRSI of cerebral gray and white matter. Magn. Reson. Med. 44, 401–411 10975892 10.1002/1522-2594(200009)44:3<401::aid-mrm10>3.0.co;2-w

[B45] Minati L. Aquino D. Bruzzone M. G. Erbetta A. (2010). Quantitation of normal metabolite concentrations in six brain regions by *in-vivo* 1H-MR spectroscopy. J. Med. Phys. 35, 154–163 10.4103/0971-6203.62128 20927223 PMC2936185

[B46] Moffett J. R. Ross B. Arun P. Madhavarao C. N. Namboodiri A. M. (2007). N-Acetylaspartate in the CNS: from neurodiagnostics to neurobiology. Prog. Neurobiol. 81, 89–131 10.1016/j.pneurobio.2006.12.003 17275978 PMC1919520

[B47] Namboodiri A. M. Peethambaran A. Mathew R. Sambhu P. A. Hershfield J. Moffett J. R. (2006). Canavan disease and the role of N-acetylaspartate in myelin synthesis. Mol. Cell Endocrinol. 252, 216–223 10.1016/j.mce.2006.03.016 16647192

[B48] Nie K. Zhang Y. Huang B. Wang L. Zhao J. Huang Z. (2013). Marked N-acetylaspartate and choline metabolite changes in Parkinson's disease patients with mild cognitive impairment. Parkinsonism Relat. Disord. 19, 329–334 10.1016/j.parkreldis.2012.11.012 23238068

[B49] Ozturk A. Degaonkar M. Matson M. A. Wells C. T. Mahone E. M. Horska A. (2009). Proton MR spectroscopy correlates of frontal lobe function in healthy children. AJNR Am. J. Neuroradiol. 30, 1308–1314 10.3174/ajnr.A1576 19357380 PMC2782857

[B50] Pfleiderer B. Ohrmann P. Suslow T. Wolgast M. Gerlach A. L. Heindel W. (2004). N-acetylaspartate levels of left frontal cortex are associated with verbal intelligence in women but not in men: a proton magnetic resonance spectroscopy study. Neuroscience 123, 1053–1058 10.1016/j.neuroscience.2003.11.008 14751296

[B51] Pouwels P. J. Brockmann K. Kruse B. Wilken B. Wick M. Hanefeld F. (1999). Regional age dependence of human brain metabolites from infancy to adulthood as detected by quantitative localized proton MRS. Pediatr. Res. 46, 474–485 10.1203/00006450-199910000-00019 10509371

[B52] Pouwels P. J. Frahm J. (1998). Regional metabolite concentrations in human brain as determined by quantitative localized proton MRS. Magn. Reson. Med. 39, 53–60 10.1002/mrm.1910390110 9438437

[B53] Provencher S. W. (2001). Automatic quantitation of localized *in vivo* 1H spectra with LCModel. NMR Biomed. 14, 260–264 10.1002/nbm.698 11410943

[B54] Provencher S. W. (2008). LCModel & LCMgui User's Manual: LCModel 6.2-1. Available online at: http://s-provencher.com/pages/lcm-manual.shtml

[B55] Rango M. Arighi A. Biondetti P. Barberis B. Bonifati C. Blandini F. (2007). Magnetic resonance spectroscopy in Parkinson's disease and parkinsonian syndromes. Funct. Neurol. 22, 75–79 Available online at: http://www.functionalneurology.com/index.php?PAGE=articolo_dett&id_article=2284&ID_ISSUE=244 17637209

[B56] Ross A. J. Sachdev P. S. (2004). Magnetic resonance spectroscopy in cognitive research. Brain Res. Brain Res. Rev. 44, 83–102 10.1016/j.brainresrev.2003.11.001 15003387

[B57] Ross A. J. Sachdev P. S. Wen W. Valenzuela M. J. Brodaty H. (2005). Cognitive correlates of 1H MRS measures in the healthy elderly brain. Brain Res. Bull. 66, 9–16 10.1016/j.brainresbull.2005.01.015 15925139

[B58] Safriel Y. Pol-Rodriguez M. Novotny E. J. Rothman D. L. Fulbright R. K. (2005). Reference values for long echo time MR spectroscopy in healthy adults. Am. J. Neuroradiol. 26, 1439–1445 Available online at: http://www.ajnr.org/content/26/6/1439.long 15956513 PMC8149071

[B59] Shim T. S. Lee J. H. Kim S. Y. Lim T. H. Kim S. J. Kim D. S. (2001). Cerebral metabolic abnormalities in COPD patients detected by localized proton magnetic resonance spectroscopy. Chest 120, 1506–1513 10.1378/chest.120.5.1506 11713127

[B60] Tabachnik B. G. Fidell L. S. (2001). Using Multivariate Statistics. London: Pearson Education

[B61] Targosz-Gajniak M. G. Siuda J. S. Wicher M. M. Banasik T. J. Bujak M. A. Augusciak-Duma A. M. (2013). Magnetic resonance spectroscopy as a predictor of conversion of mild cognitive impairment to dementia. J. Neurol. Sci. 335, 58–63 10.1016/j.jns.2013.08.023 24035276

[B62] Tran T. Ross B. Lin A. (2009). Magnetic resonance spectroscopy in neurological diagnosis. Neurol. Clin. 27, 21–60, xiii. 10.1016/j.ncl.2008.09.007 19055974

[B63] Valenzuela M. J. Sachdev P. S. Wen W. Shnier R. Brodaty H. Gillies D. (2000). Dual voxel proton magnetic resonance spectroscopy in the healthy elderly: subcortical-frontal axonal N-acetylaspartate levels are correlated with fluid cognitive abilities independent of structural brain changes. Neuroimage 12, 747–756 10.1006/nimg.2000.0629 11112406

[B64a] Wang Y. Li S. J. (1998). Differentiation of metabolic concentrations between gray matter and white matter of human brain by *in vivo* 1H magnetic resonance spectroscopy. Magn. Reson. Med. 39, 28–33 10.1002/mrm.1910390107 9438434

[B64] Wechsler D. (1997a). Wechsler Abbreviated Scale of Intelligence. San Antonio, TX: The Psychological Corporation

[B65] Wechsler D. (1997b). Wechsler Adult Intelligence Scale-III. San Antonio, TX: The Psychological Corporation

[B66] Wiedermann D. Schuff N. Matson G. B. Soher B. J. Du A. T. Maudsley A. A. (2001). Short echo time multislice proton magnetic resonance spectroscopic imaging in human brain: metabolite distributions and reliability. Magn. Reson. Imag. 19, 1073–1080 10.1016/S0730-725X(01)00441-6 11711231

[B67] Yeo R. A. Hill D. Campbell R. Vigil J. Brooks W. M. (2000). Developmental instability and working memory ability in children: a magnetic resonance spectroscopy investigation. Dev. Neuropsychol. 17, 143–159 10.1207/S15326942DN1702_01 10955200

